# Taxonomic Significance of Leaf Anatomical Characteristics of Selected *Globba* L. (Zingiberaceae) Species in Peninsular Malaysia

**DOI:** 10.21315/tlsr2025.36.2.3

**Published:** 2025-07-31

**Authors:** Noraini Talip, Syazwani Basir, Che Nurul Aini Che Amri, Mohd. Norfaizal Ghazalli, Ahmad Fitri Zohari, Muhammad Amirul Aiman Ahmad Juhari, Mohamad Ruzi Abdul Rahman, Hamidun Bunawan

**Affiliations:** 1 Department of Biological Sciences and Biotechnology, Faculty of Science and Technology, Universiti Kebangsaan Malaysia, 43600 UKM Bangi, Selangor, Malaysia; 2 Department of Plant Science, Kulliyyah of Science, International Islamic University Malaysia Kuantan Campus, 25200 Kuantan, Pahang, Malaysia; 3 Programme of Resource Utilisation and Agrobiodiversity Conservation, Agrobiodiversity and Environment Research Centre, MARDI, 43400 Serdang, Selangor, Malaysia; 4 Department of Environment, Faculty of Forestry and Environment, Universiti Putra Malaysia, 43400 UPM Serdang, Selangor, Malaysia; 5 Bangi Botanic Gardens, Faculty of Science and Technology, Universiti Kebangsaan Malaysia, 43600 UKM Bangi, Selangor, Malaysia; 6 Institute of Systems Biology, Universiti Kebangsaan Malaysia, 43600 UKM Bangi, Selangor, Malaysia

**Keywords:** Zingiberaceae, *Globba*, *Globba variabilis* var. *pusilla*, Leaf Anatomy, Peninsular Malaysia, Zingiberaceae, *Globba*, *Globba variabilis* var. *pusilla*, Anatomi Daun, Semenanjung Malaysia

## Abstract

A comparative leaf anatomy study was carried out on 10 taxa of *Globba* L. (Zingiberaceae) in Peninsular Malaysia to ascertain their systematic significance, especially for species differentiation and identification. Methods used include leaf clearing, leaf sectioning using a sliding microtome and observation under a scanning electron microscope. Results showed that a combination of the following characters have taxonomic significance in *Globba* spp. Studied, i.e., outlines of leaf margin, midribs, petioles and their relative sizes; number of vascular bundles in arc I, II, III and IV in the midribs and petioles; absence or presence and type of trichomes; presence of fibre caps and girders in vascular bundles in the leaf lamina, as well as the presence of hypodermal layers below adaxial epidermis. Anatomical characteristics such as tufted trichomes in *G. patens*, papilose adaxial epidermal cells in *G. variabilis* var. *pusilla*, rounded margin in *G. variabilis* and an arc I vascular bundle present only in the midrib of *G. variabilis* var. *pusilla* can be used as diagnostic characters for each species. *Globba variabilis* var. *pusilla* which is closely related to *G. variabilis* can be distinguished by the papilose epidermal cells. This study proved that leaf epidermis anatomical characteristics could possibly be applied to the identification of some species in *Globba* with certainty.

## Abstrak

Kajian perbandingan anatomi daun telah dijalankan ke atas 10 takson *Globba* L. (Zingiberaceae) di Semenanjung Malaysia untuk menentukan kesignifikan nilai taksonomi terutamanya untuk pembezaan dan pengenalpastian spesies. Kaedah yang digunakan termasuk pembersihan dan hirisan daun menggunakan mikrotom gelongsor serta pemerhatian di bawah mikroskop imbasan elektron. Hasil kajian menunjukkan bahawa gabungan ciri anatomi mempunyai nilai taksonomi bagi spesies *Globba* yang dikaji, iaitu ciri bentuk luaran petiol, tepi dan tulang daun, dan saiznya secara relatif; berkas vaskular tersusun dalam bentuk arka mengikut sistem I, II, III dan IV pada petiol dan tulang daun; kehadiran dan ketidakhadiran trikom; kehadiran kelompok dan tiang sel serabut pada berkas vaskular lamina daun, dan juga kehadiran lapisan sel hipodermis di bawah epidermis adaksial. Ciri anatomi seperti kehadiran trikom berumpun pada *G. patens*, sel epidermis adaksial berpapila pada *G. variabilis* var. *pusilla*, berkas vaskular membulat hanya ditemui pada *G. variabilis* dan arka I sahaja ditemui hanya pada tulang daun *G. variabilis* var. *pusilla*, boleh digunakan sebagai ciri diagnostik. *Globba variabilis* var. *pusilla* yang berkait rapat dengan *G. variabilis* boleh dibezakan oleh sel epidermis berpapila. Kajian ini membuktikan bahawa ciri anatomi epidermis daun mempunyai nilai taksonomi dan boleh digunakan untuk pengecaman beberapa spesies *Globba* yang dikaji.

**Highlights**

- A comparative leaf anatomy on 10 taxa of *Globba* L. (Zingiberaceae) in Peninsular Malaysia to ascertain their systematic significance, especially for species differentiation and identification.
- A combination of leaf anatomical characteristics that have systematic significance in *Globba* spp. studied especially in species differentiation and identifying certain species such as tufted trichomes in *G. patens*, and papilose adaxial epidermal cells in *G. variabilis* var. *pusilla. Globba variabilis* var. *pusilla* which is closely related to *G. variabilis* can be distinguished by the papilose epidermal cells.
- This study proved that trichomes evidence could be applied to the identification of species in *Globba* with certainty.

## INTRODUCTION

*Globba* L. is a genus of tribe Globbeae, which belongs to the subfamily Zingiberoideae from family Zingiberaceae. It is found in various regions including India, Sri Lanka, southern China, Southeast Asia and northern Australia (Sangvirojnapat *et al*. 2019). The main area of distribution in monsoonal Southeast Asia, particularly in Thailand and Myanmar (Kress *et al*. 2002; Gowda *et al.* 2012). Globbeae comprises the genera *Globba*, *Gagnepainia, Hemiorchis* and *Mantisia*, which are small and belong to one of the two tribes of the subfamily. The other three genera of Globbae have a more limited distribution and are entirely encompassed within the range of *Globba* itself (Williams *et al.* 2004, Schumann 1904; Holttum 1950). *Gagnepainia* is predominantly located in Thailand, Laos, Vietnam and Cambodia, whereas *Hemiorchis* and *Mantisia* are distributed in northeastern India, Myanmar and Bangladesh (Williams *et al.* 2004).

An investigation was carried out using parsimony and Bayesian analyses on nuclear internal transcribed spacer (ITS) and plastid trnK-matK data. The data was obtained from a wide range of samples belonging to the *Globba* and other related genera. The results indicate that *Mantisia* is a monophyletic that is nested inside *Globba.* Additionally, *Hemiorchis* and *Gagnepainia* are monophyletic genera that are sister to each other (Williams *et al.* 2004). The number and shape of the anther’s appendages, as well as the morphology of its inflorescence and fruits, are crucial characteristics for comprehending the evolutionary relationships within the *Globba* species (Iwamoto *et al.* 2020; Williams *et al.* 2004)

The conventional infrageneric classification in *Globba* has primarily emphasised the number of anther appendages, which can be zero, two or four (Schumann 1904; Williams *et al.* 2004). Based on this, the genus was classified into three sections as follows (Cao *et al.* 2019):

1. *Globba* sect. *Globba* which has four anther appendages.
2. *Globba* sect. *Haplanthera (*Horan.) without anther appendages.
3. *Globba* sect. *Ceratanthera* (Horan.) Petersen which has two anther appendages.

Williams *et al.* (2004) published the findings of a molecular phylogenetic investigation on *Globba* using ITS and *trnK-matK* nucleotide sequence data. The data indicated that *Globba* should be classified into three subgenera and seven sections. Three well-supported, monophyletic groups were recently identified, namely *Globba* sect. *Mantisia, Globba* sect. *Substrigosa* and *Globba* sect. *Sempervirens*. Additionally, the group previously known as *Globba* (sect. *Ceratanthera*) series *Mediocalcaratae* was reclassified as *Globba* (sect. *Nudae*) subsect. *Mediocalcaratae* (Williams *et al.* 2004).

*Globba* is the third largest genus of the Zingiberaceae, which consists of 55 genera and over 1,300 species (Akram *et al.* 2023). It is surpassed in terms of species number only by the polyphyletic genera *Alpinia* and *Amomum* (Mabberley 2017; Kress *et al.* 2002). Occasionally, new species are being documented, leading to a gradual increase in the total number of species. Sunisa *et al.* (2022) have recently identified and described ten new species of *Globba* in continental Southeast Asia.

Most species are medium-sized herbaceous plants that grow in semi-shaded areas, either as terrestrial plants or lithophytes. *Globba* can be distinguished from the other genera of Zingiberaceae by its long exserted filament, its distinctive flower with a fused lip and stamen, and the long-exserted and curved stamen. There are 750 species in Malaysia that belong to 31 genera (Govaerts *et al.* 2022). Meanwhile, Peninsular Malaysia has reported about 200 species from 19 genera (Nagappan *et al.* 2019; Akram *et al.* 2023). Within all species found in Peninsular Malaysia, the anther is spurred with either one or two triangular appendages along the margin, a unique characteristic of this genus (Sam *et al.* 2016; Larsen *et al.* 1999).

Some species, such as *Globba variabilis* var. *pusilla* with dark green, velvety leaves and orange flowers, possess significant ornamental value. The *Globba atrosanguiena* Teysm. & Binn., a species found in the wild in Sabah (Danum Valley), has already significantly gained popularity due to its attractiveness as a potted plant. Despite the species are abundant in Malaysia, there is still a scarcity of anatomical study on them. The only study was conducted by Tomlinson (1956), but only on a select few species. Species identification within the genus is frequently challenging due to hybridisation between different species (Shi *et al.* 2011). Consequently, alternative characteristics are required for species identification.

The objective of this study is to investigate whether vegetative anatomy could be used as additional data in the identification of the *Globba* species. This study was also undertaken to investigate the potential of leaf anatomy in identifying species in the absence of flowers and to broaden the understanding of the anatomical characteristics of Zingiberaceae. Previous studies on leaf anatomy in specific genera in Zingiberaceae, such as *Alpinia* in China (Hussin *et al.* 2000), *Alpinia* in Malaysia (Noraini *et al.* 2005), Zingiber (Zhao *et al.* 2022), *Globba* in Thailand (Kajornjit *et al.* 2018), *Boesenbergia* and *Kaempferia* (Hussin *et al*. 2000), have demonstrated that a combination of anatomical features can be valuable for identifying species and potentially for classification purposes.

## MATERIALS AND METHODS

Fresh leaf materials for anatomical study were obtained from several forest reserves in Peninsular Malaysia and were mainly used except in a few cases where small, dried leaf samples were obtained from Universiti Kebangsaan Malaysia Herbarium (UKMB), Universiti Kebangsaan Malaysia, Bangi, Selangor, Malaysia. Voucher specimens have been deposited in the UKMB, Bangi, Selangor, Malaysia. Fresh specimens used in this study were obtained from the nursery at the School of Biological Sciences, Universiti Malaya, Rimba Ilmu, Universiti Malaya and Taman Negara Merapoh, Pahang. Ten *Globba* species with several replicates were employed in this study. The voucher specimens were deposited at the Herbarium of Universiti Kebangsaan Malaysia, Malaysia for future reference. The study’s species list is shown in Table 1.

The process of fixation, embedding and sectioning was carried out according to the method described by Johansen (1940) and Sass (1958), with certain modifications as outlined by Noraini *et al.* (2019). Fresh materials were fixed in A:A (1:3), of 25% acetic acid and 70% ethanol. Dried herbarium materials were boiled, then fixed using the same solution. Leaf specimens were sectioned with a sliding microtome at 20 μm–30 μm thickness and stained in 1% Safranin in 50% alcohol and 1% Alcian Green in 100 mL purifying water with three drops of acetic acid. Sections were made from the middle and marginal parts of the leaf lamina using a Reichert sliding microtome (manufactured in Germany). Epidermal peels were prepared by mechanical scraping and stained in Safranin. For venation studies, leaves were cleared in 70% alcohol with a drop of hydrochloric acid, dehydrated and stained in 1% Basic Fuchsin in 6% KOH. All slides were mounted in Euparal after dehydration. Photomicrographs of sections and epidermal peels were made using either a Leitz Diaplan polarising microscope fitted with a JVC CCD camera or a Reichert Polyvar 2 microscope fitted with a digital camera. Images were processed using Analysis Docu Software (soft-imaging system). All slides were deposited in the anatomy section at the Microtechnique Laboratory, Universiti Kebangsaan Malaysia.

The micromorphological structures were examined using scanning electron microscopy (SEM). Initially, the samples were cut into 0.5 cm × 0.5 cm on a wax plate (Cavex) to protect the samples (Basir *et al.* 2022). Subsequently, the samples underwent a triple rinse in a 0.1 M phosphate buffer solution (PBS) with a pH of 7.4 for a duration of 10 min. The dehydration was conducted using a series of ethanol solutions (35%, 50%, 70%, 80%, 90% and 99%) for 10 min per concentration. For the 99% ethanol solution, the dehydration process was repeated three times (Wiemer *et al.* 2009; Kowalkowska *et al.* 2015). The samples were subsequently dehydrated using critical point drying (CPD) (Leica® EM CPD300) to remove the ethanol, followed by a coating with gold for 10 min. The micromorphological structures were analysed using a SEM at magnifications ranging from 100× to 1,500×.

## RESULTS

### I. Leaf Surface Anatomical Characteristics Under LM and SEM

**Figure 1:**
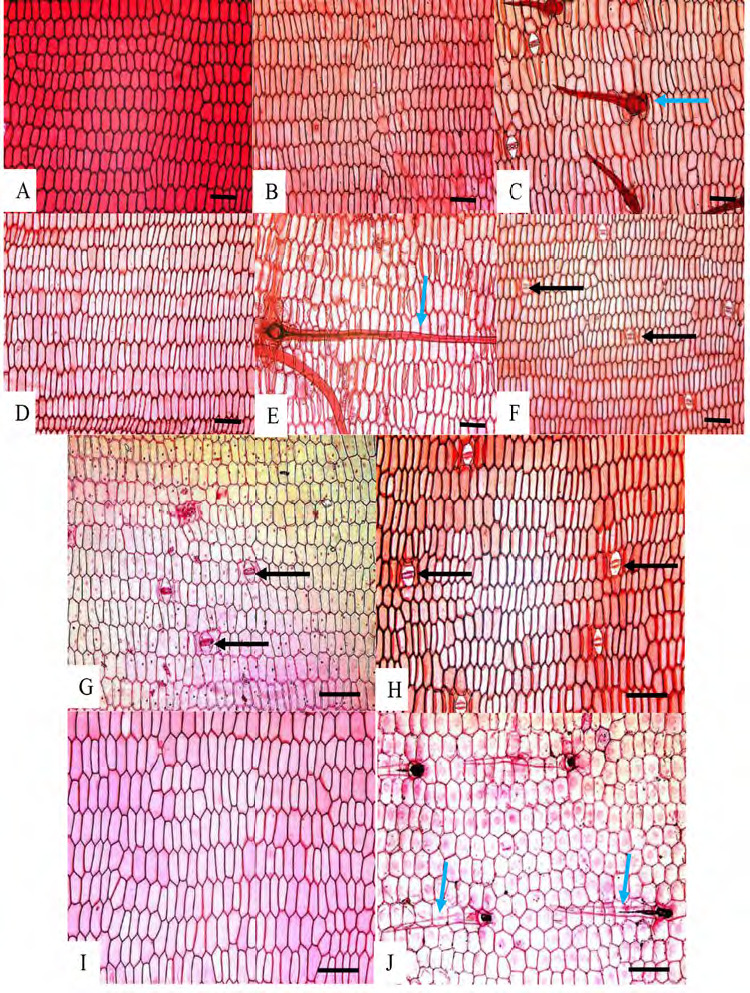
Adaxial epidermis of leaf lamina: (A) *G. aurantiaca*, (B) *G. cernua*, (C) G*. leucantha*, (D) *G. patens*, (E) *G. patens* var *costulata*, (F) *G. pendula*, (G) *G. roxburghiana*, (H) *G. unifolia*, (I) *G. variabilis*, and (J) G. *variabilis* var *pusilla.* Blue arrows indicate the presence of trichomes, while black arrows indicate the presence of stomata in the adaxial epidermis of the leaf *lamina.* Scale bar: 200 μm.

**Figure 2:**
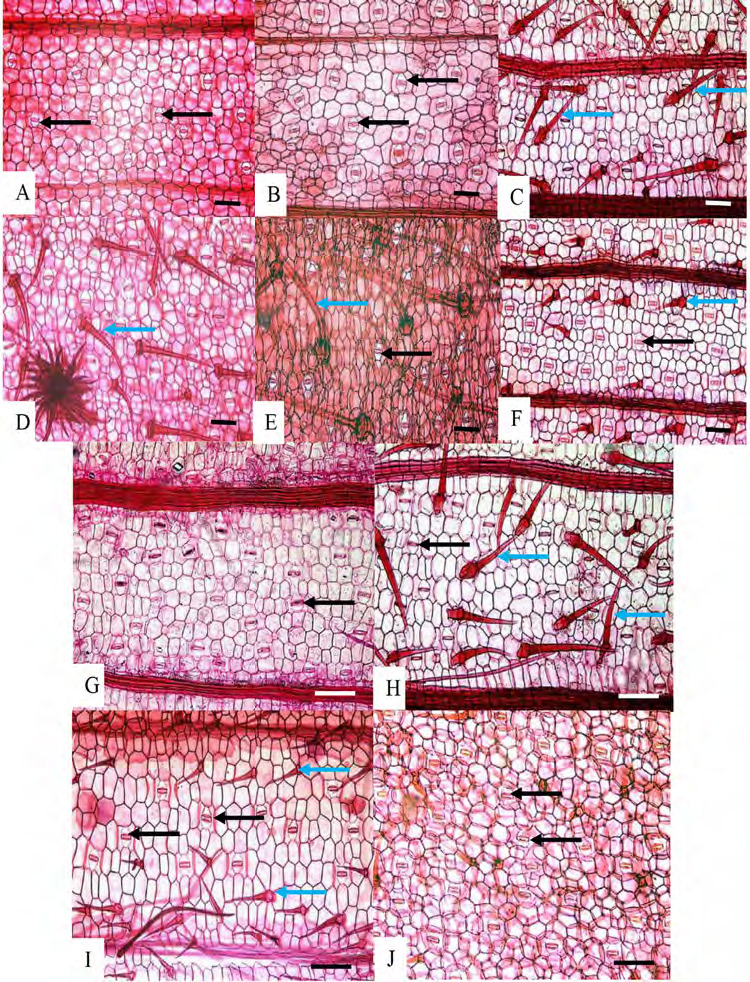
Abaxial epidermis of leaf lamina: (A) *G. aurantiaca*, (B) *G. cernua*, (C) G*. leucantha*, (D) *G. patens*, (E) *G. patens* var *costulata*, (F) *G. pendula*, (G) *G. roxburghiana*, (H) *G. unifolia*, (I) *G. variabilis*, and (J) *G. variabilis* var *pusilla.* Blue arrows indicate the presence of trichomes, while black arrows indicate the presence of stomata in the abaxial epidermis of the leaf *lamina.* Scale bar: 200 μm.

**Figure 3:**
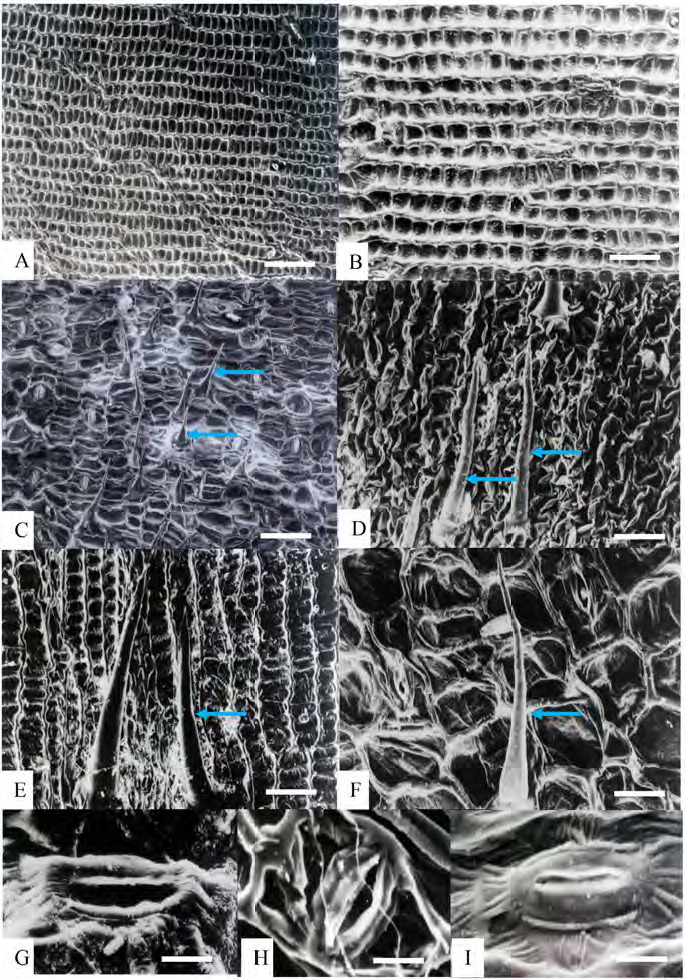
Leaf epidermis cuticle and anticlinal wall characteristics: (A) *G. cernua*, (B) *G. roxburghiana*, (C) G*. leucantha*, (D) *G. patens*, (E) *G. patens* var *costulata*, (F) *G. pendula.* Stomata: (G) *G. pendula*, (H) *G. roxburghiana*, and (I) G*. leucantha.* Blue arrows indicate the present of trichomes at leaf epidermis cuticle wall. Scale bar: A, B, C, D = 200 μm, E, F = 50 μm, G, H, I = 100 μm.

Epidermal cells: anticlinal walls straight in both abaxial and adaxial epidermal surfaces, cells rectangular and hexagonal with longer axis usually perpendicular to veins (Figs. 1 and 2). Stomata: tetracytic, four subsidiary cells, subsidiary and epidermal cells can be differentiated, elliptical in shape, guard cells at same level as epidermal cells (Figs. 3G, 3H, 3I), long axis of pore parallel to veins on abaxial epidermis, present randomly scattered between veins in all species studied, more frequent on the abaxial epidermis and very rare on adaxial epidermis; amphistomatic (stomata occurs in both abaxial and adaxial leaf surface) in four species, *G. leucantha, G. pendula, G. roxburghiana* and *G. unifolia* (Figs. 1C, 1F, 1G, 1H); hypostomatic (stomata occurs only on abaxial leaf surface) in six taxa, *G. aurantiaca, G. cernua, G. patens, G. patens* var. *costulata, G. variabilis* and *G. variabilis* var. *pusilla*. Trichomes: Two types of trichomes were present; simple, unicellular trichomes and tufted trichomes. Simple, unicellular trichomes were present on adaxial epidermis of *G. leucantha* (Fig. 1C), *G. patens* var. *costulata* (Fig. 1E), and *G. variabilis* var. *pusilla* (Fig. 1J), and on the abaxial epidermis of *G. leucantha, G. patens, G. patens* var. *costulata, G. pendula, G. unifolia, G. variabilis* and *G. variabilis* var. *pusilla* (Figs. 2C, 2D, 2E, 2F, 2H, 2I, 2J). Whereas tufted trichomes are present only on the abaxial epidermis of *G. patens* (Fig. 2D).

### II Leaf Lamina Anatomical Characteristics under LM

**Figure 5:**
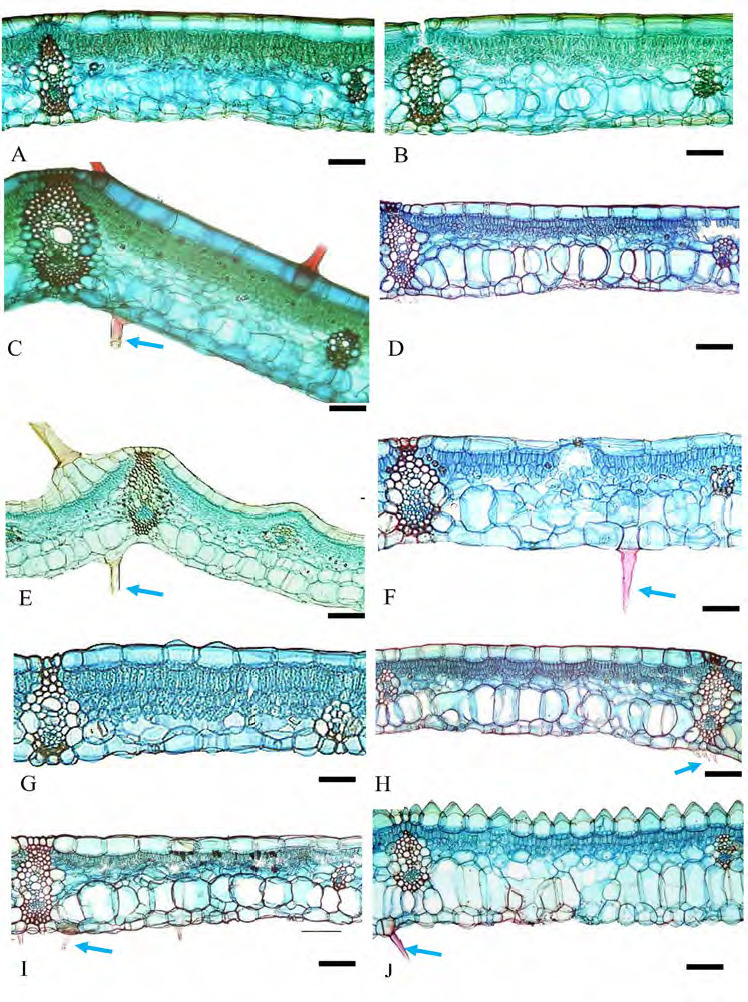
Cross section of leaf lamina: (A) *G. aurantiaca*, (B) *G. cernua*, (C) G*. leucantha*, (D) *G. patens*, (E) *G. patens* var *costulata*, (F) *G. pendula*, (G) *G. roxburghiana*, (H) *G. unifolia*, (I) *G. variabilis*, and (J) *G. variabilis* var *pusilla.* Blue arrows indicate the presence of trichomes. Scale bar: 200 μm.

Epidermal cells: adaxial epidermal cells long, length twice than width, abaxial epidermal cells slightly similar in size but compressed. Papillose epidermal cells are present only in *G. variabilis* var. *pusilla* (Fig. 5J). Hypodermis: one continuous layer, present abaxially in all species studied (Figs. 5A–5J). Chlorenchyma: palisade cells in 1–3 layers, the second or third layer smaller and wider and resembling mesophyll cells, sometimes making it indistinguishable; spongy cells in 2–3 layers. Vascular bundles: collateral bundles with tracheary elements consisting of 1–2 metaxylem elements and a few protoxylem cells flanked by colourless parenchyma laterally (Figs. 5A–5J). Fibre cells usually form abaxial and adaxial caps in main or large vascular bundles and extend to abaxial epidermal cells in all taxa studied except in *G. variabilis* var. *pusilla* (Fig. 5J). In large vascular bundles, fibres also extend as girders to the adaxial epidermis in all species studied except in *G. aurantiaca, G. cernua* and *G. variabilis* var. *pusilla* (Figs. 5A, 5B and 5J); in smaller bundles fibres form only as an abaxial caps in *G. aurantiaca, G. cernua, G. patens* var*. costulata, G. pendula and G. roxburghiana* and *G. variabilis* (Figs. 5A, 5B, 5E, 5F, 5G and 5I), whereas fibres form as an abaxial and adaxial caps in *G. leucantha, G. patens, G. unifolia*, and *G. variabilis* var. *pusilla* (Figs. 5C, 5D, 5H and 5J); smaller vascular bundles situated in equidistant between adaxial and abaxial epidermis with fibre caps touching the hypodermis layer except in *G. roxburghiana*, fibre cap touching abaxial epidermis (Fig. 5G). Girders 2 to 6 cells wide, are present in large bundles only. Crystals: solitary crystals present in mesophyll cells of all species investigated (Figs. 5A–5J).

### III. Leaf Margin Anatomical Characteristics under LM

**Figure 6:**
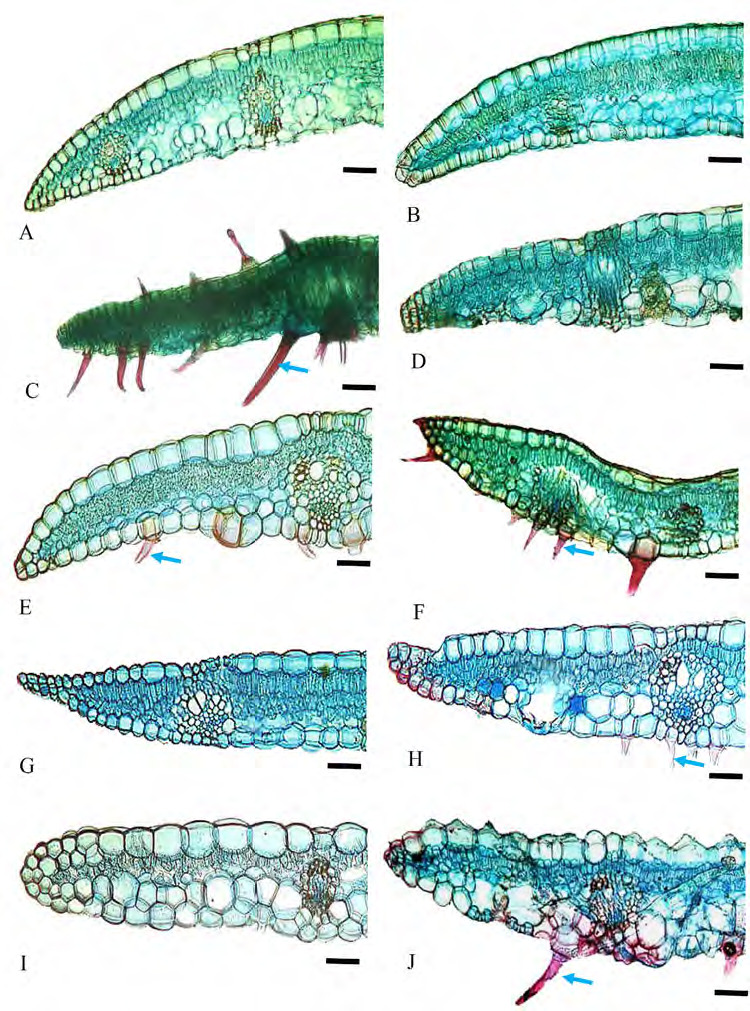
Cross section of marginal leaf: (A) *G. aurantiaca*, (B) *G. cernua*, (C) *G. leucantha*, (D) *G. patens*, (E) *G. patens* var *costulata*, (F) *G. pendula*, (G) *G. roxburghiana*, (H) *G. unifolia*, (I) *G. variabilis*, and (J) *G. variabilis* var *pusilla.* Blue arrows indicate the presence of trichomes. Scale bar: 200 μm.

Outline shape: tapering except rounded in *G. variabilis*. The tip of the margin can be short, long, straight, recurved downward and recurved upward. The tip of margin is longer in *G. roxburghiana* (Fig. 6G), straight in *G. unifolia, G. variabilis* and *G. variablis* var. *pusilla* (Figs. 6H, 6I & 6J), recurved down in *G. aurantiaca, G. cernua, G. leucantha, G. patens* and *G. patens* var. *costulata* (Figs. 6A, 6B, 6C, 6D and 6E), recurved upwards in *G. pendula*, and *G. roxburghiana* (Figs. 6F and 6G). Portion beyond last bundle: consisting of mesophyll cells, except in *G. variabilis* where portion beyond last bundle consisting or colourless parenchyma (Fig. 6I).

### IV. Midrib Anatomical Characteristics under LM

**Figure 7:**
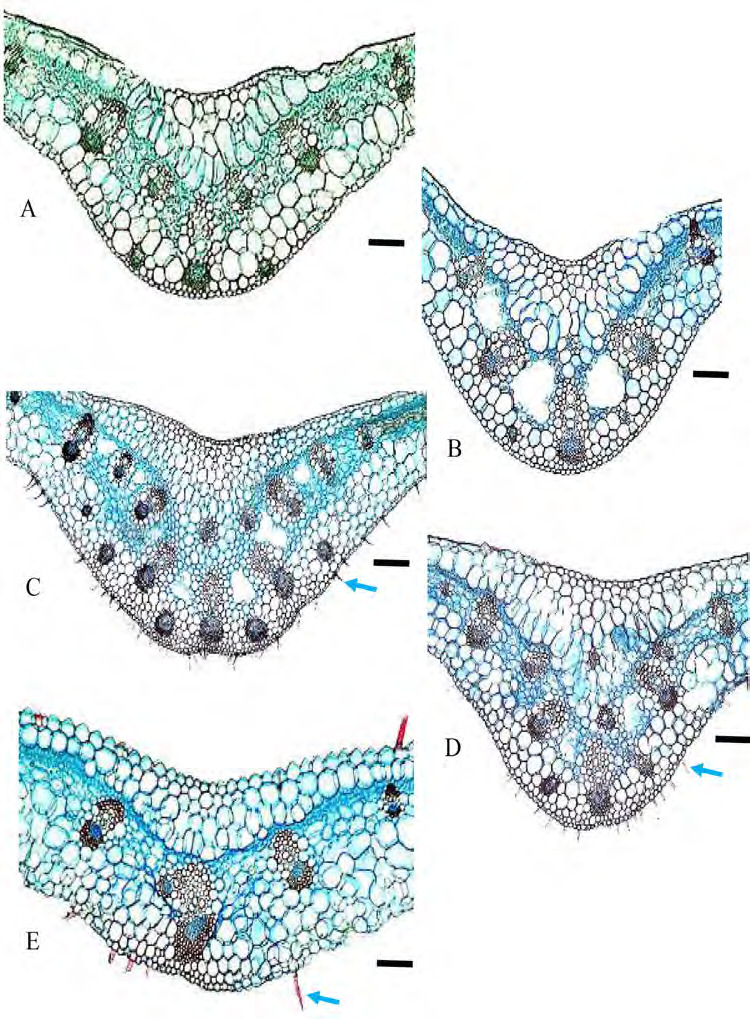
Cross section of midribs: (A) *G. pendula*, (B) *G. roxburghiana*, (C) *G. unifolia*, (D) *G. variabilis*, and (E) *G. variabilis* var. *pusilla.* Blue arrows indicate the presence of trichomes. Scale bar: 200 μm.

Outline shape: adaxial surface more or less curved, abaxial surface arched (Fig. 7). Trichomes: simple unicellular in all species examined but absent in *G. pendula* (Fig. 7A) and *G. roxburghiana* (Fig. 7B). Collenchyma: absent. Vascular bundle: vascular bundles arranged in several arcs; main arc is described as arc 1 (large bundles, near to the abaxial epidermis), arc II (smaller bundles, subsidiary arc near to the abaxial epidermis), arc III (smaller bundles, subsidiary arc above the main arc or arc I), and arc IV (smaller bundles, subsidiary arc closer to the adaxial epidermis). Arc I only in *G. variabilis* var. *pusilla* (Fig. 7E), arc 1 and II in *G. patens* var. *costulata* and *G. pendula* (Fig. 7A), arc I, II and III in *G. aurantiaca, G. cernua, G. leucantha, G. patens*, *G. patens* var. *costulata*, *G. roxburghiana* (Fig. 7B), *G. unifolia* (Fig. 7C) and *G. variabilis* (Fig. 7D). Bundles consist of one metaxylem cells with several protoxylem cells flanked by parenchyma cells lacking chloroplasts on either side; fibres form caps adaxially and abaxially. Fibre cells are not in contact with abaxial epidermis, occurring one to few cells below it. Crystals: Solitary in parenchyma cells of all species studied.

### V. Petiole Anatomical Characteristics under LM

**Figure 8:**
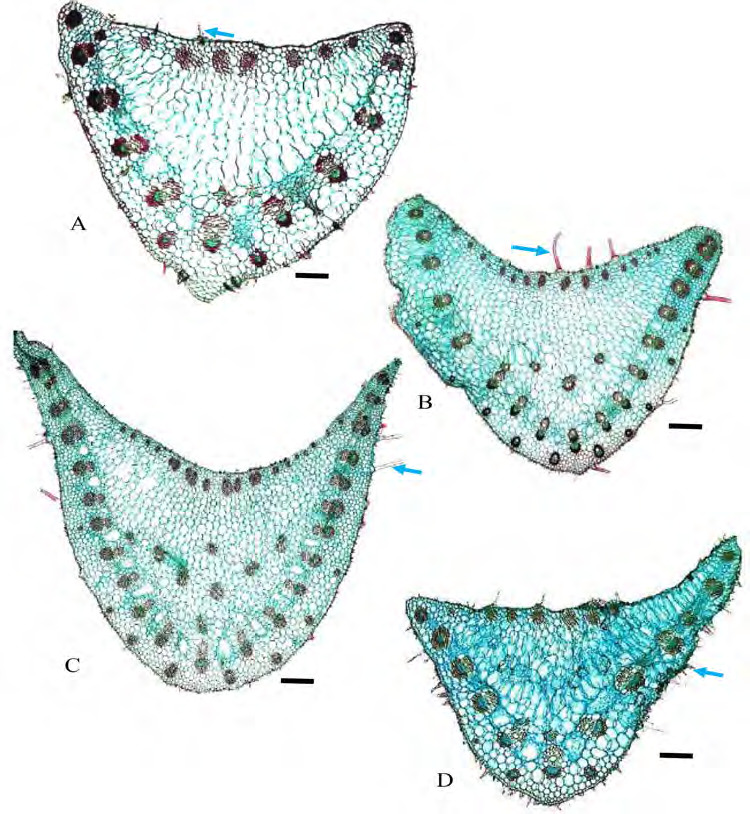
Cross section of petiole: (A) *G. aurantiaca*, (B) *G. cernua*, (C) G*. leucantha*, and (D) *G. patens.* Blue arrows indicate the present of trichomes. Scale bar: 200 μm.

**Figure 9:**
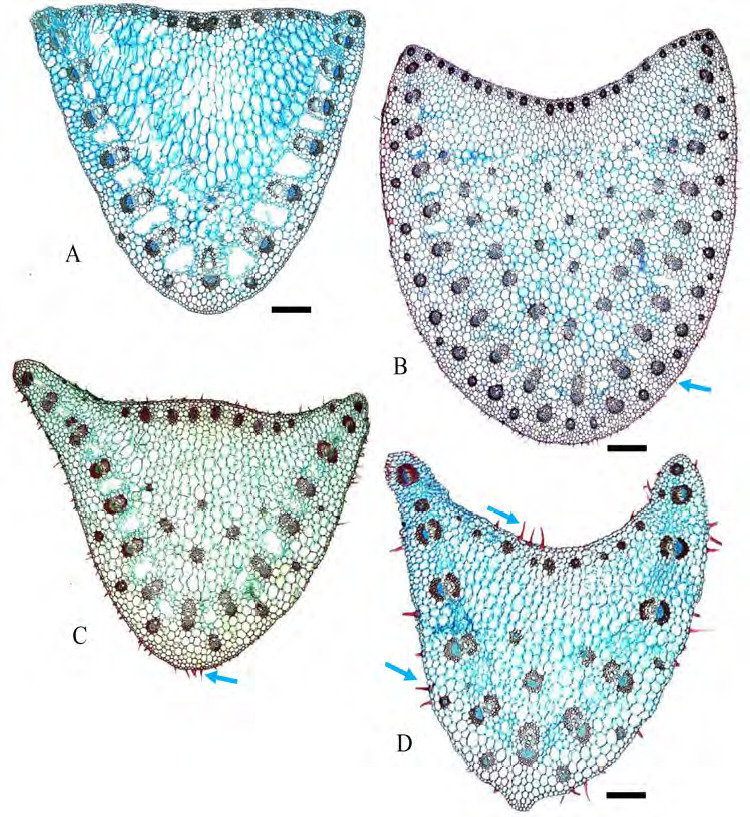
Cross section of petioles: (A) *G. roxburghiana*, (B) *G. unifolia*, (C) G*. variabilis*, and (D) *G. variabilis* var *pusilla.* Blue arrows indicate the present of trichomes. Scale bar: 200 μm.

Outline shape: adaxial surface almost straight in *G. roxburghiana* (Fig. 9A), convex in *G. variabilis* (Fig. 9C), more or less concave in most taxa studied (Figs. 8 and 9), and ends extended into wings in *G. patens* var. *costulata*; abaxial surface arched or wide V-shaped in most taxa observed, except U-shaped in *G. unifolia* (Fig. 9B). Collenchyma: absent. Vascular bundles: vascular bundles arranged in several arcs; description follows Tomlinson (1956); main arc is described as arc 1, abaxial arc as arc II, adaxial arc as arc III and a fourth arc closer to the adaxial epidermis as arc IV. All species consists of arcs I, II, III and IV (Figs. 8 and 9). Bundles of arcs II, III and IV are usually smaller than bundles in arc I, sometimes are very small particularly bundles in arc IV. The number of bundles in arcs I, II, III and IV are varies depending on the size of petiole (Table 2). Arc IV bundles smaller than arc III bundles and often consisting of fibres only. In all species observed arc III and IV bundles are well separated. Width of outer layer tissue between bundle and abaxial epidermis varies between 1 to 4 cells and all species arc II bundles do not connect to abaxial epidermis. Air lacunae present between arc I bundles in all species and more obvious in *G. roxburghiana* (Fig. 9A). Trichomes: Two types of trichomes were present; simple, unicellular trichomes and tufted trichomes. Simple, unicellular trichomes were present on adaxial epidermis of *G. leucantha* (Fig. 1C), *G. patens* var. *costulata* (Fig. 1E) and *G. variabilis* var. *pusilla* (Fig. 1J), and on the abaxial epidermis of *G. leucantha, G. patens, G. patens* var. *costulata, G. pendula, G. unifolia, G. variabilis* and *G. variabilis* var. *pusilla* (Figs. 2C, 2D, 2E, 2F, 2H and 2I). Whereas tufted trichomes are present only on the abaxial epidermis of *G. patens* (Fig. 2D). Table 2 presents a summary of the anatomical characteristics of the midrib and petiole, whereas Table 3 provides a summary of the leaf lamina of the species studied. Table 4 shows the dichotomous identification key constructed, including the atomical characteristics of the studied *Globba* species.

## DISCUSSION

The significant variations observed included: (a) vascular system and the number of vascular bundles in leaf midrib and petiole; (b) petiole outline; (c) tip of leaf margin, either long, short, recurved upward or recurved downward; and (d) stomata occurrence (amphistomatic and hypostomatic). The combination of all the interspecies anatomical variation as above can be used to differentiate all ten taxa in this study.

The vascular system in leaf midrib and petiole of *Globba* were based on descriptions by Tomlinson (1956) that documented arc I–IV occurred in both midrib and petiole. Kajornjit *et al.* (2018) also identified arc I–IV in midrib of *Globba* in Thailand. However, in this study, only arc I–III were observed in midrib and arc I–IV in petiole. The number of vascular bundles in each arc in both midrib and petiole of *Globba* in this study are very useful for species identification and can be used to differentiate each taxon studied (Table 2). The character was first described in detail in this paper for *Globba* in Malaysia.

Khatijah *et al.* (2000) found that interspecific variation occurs in the shape of petiole in *Alpinia* (Zingiberaceae). In this study, variations in the petiole outline gives a significant difference between *G. patens* var. *costulata, G. roxburghiana, G. unifolia* and *G. variabilis* to other taxa studied. Most taxa had concave adaxial surface, whereas *G. roxburghiana* exhibited straight adaxial surface and *G. variabilis* exhibited convex adaxial surface. *G. patens* var. *costulata* on the other hands, exhibited wing-like structure on extended end of petiole.

Four types of margin tip were observed in this study. *G. roxburghiana* could easily be differentiate from other taxa by having a longer margin tip. *G. unifolia, G. variabilis* and *G. variablis* var. *pusilla* had straight margin tip, recurved downwards in *G. aurantiaca, G. cernua, G. leucantha, G. patens* and *G. patens* var. *costulata*, and recurved upwards in *G. pendula*, and *G. roxburghiana*. The recurved downward type of margin tip for the genus *Globba* was first reported by Kajornjit *et al.* (2018) in *G. candida*, a species from Thailand.

Stomata occurrences are very useful for taxonomic study of *Justicia* (Acanthaceae) (Amirul-Aiman *et al.* 2019). In this study, amphistomatic stomata only occurs in four species, which is *G. leucantha, G. pendula, G. roxburghiana* and *G. unifolia*, while the others are hypostomatic. Kajornjit *et al.* (2018) reported only amphistomatic stomata occurred in the Thailand’s *Globba*.

## CONCLUSION

The findings of the present study have shown about four common characteristics in all taxa studied, in agreement with the studies by Tomlinson (1956; 1960) for the family and genus. Such characteristics are as follows:

1. Type of stomata.
2. The presence of simple, unicellular trichome.
3. The presence of hypodermal cells in one continuous layer on the abaxial leaf surface.
4. Midrib outline.
5. The presence of solitary crystals in the mesophyll of lamina transverse section.

Hussin *et al.* (2000) also reported that the same type of trichomes and the presence of solitary crystals are common for genus *Alpinia* (Zingiberaceae) in China. Thus, these five significant characters could be used to group all taxa studied under the same genus *Globba.*

Diagnostic anatomical characteristics such as tufted trichomes in *G. patens*, rounded margin in *G. variabilis* and only arc I vascular bundle present in midrib of *G. variabilis* var. *pusilla* can be used to differentiate each taxon. *Globba variabilis* var. *pusilla* which is closely related to *G. variabilis* can also be distinguished from other species based on the papillose epidermal cells. Other diagnostic leaf anatomical characteristics are straight adaxial surface in *G. roxburghiana*, U-shaped in *G. unifolia* and convex in *G. variabilis* petiole’s outline shape, and also ends extended into wings in *G. patens* var. *costulata*. The results showed that combinations of all anatomical characteristics reported in this study will help in identification of *Globba* species studied.

## Figures and Tables

**Figure 4:**
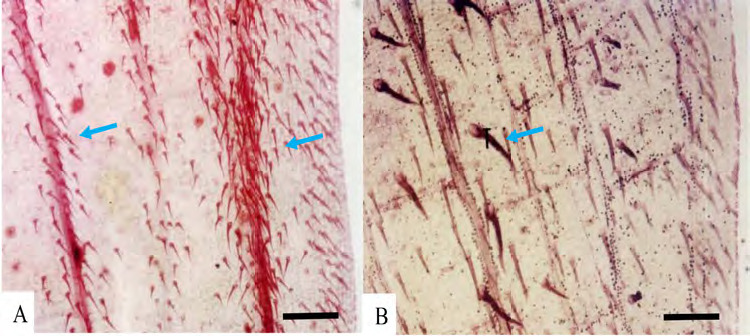
Leaf margin characteristics of some species studied, showing present of trichomes and pattern of leaf margin venation: (A) *G. unifolia*, and (B) *G. variabilis.* Blue arrows indicate the presence of trichomes. Scale bar: 200 μm.

**Table 1:** The list of species, ID number and locality species studied.

Species	ID number	Locality	Collector
*Globba aurantiaca* Miq.	NTZ1	Rimba Ilmu, Universiti Malaya, Kuala Lumpur	Noraini Talip
	NTZ2	Nursery of School of Biological Sciences, Universiti Malaya	Noraini Talip
*Globba cernua* Baker.	NTZ3	Rimba Ilmu, Universiti Malaya, Kuala Lumpur	Noraini Talip
	NTZ4	Nursery of School of Biological Sciences, Universiti Malaya	Noraini Talip
*Globba leucantha* Miq.	NTZ5	Rimba Ilmu, Universiti Malaya, Kuala Lumpur	Noraini Talip
	NTZ6	Nursery of School of Biological Sciences, Universiti Malaya	Noraini Talip
*Globba patens* Miq	NTZ7	Rimba Ilmu, Universiti Malaya, Kuala Lumpur	Noraini Talip
	NTZ8	Nursery of School of Biological Sciences, Universiti Malaya	Noraini Talip
*Globba patens* var. *costulata* Lim.	NTZ9	Rimba Ilmu, Universiti Malaya, Kuala Lumpur	Noraini Talip
	NTZ10	Nursery of School of Biological Sciences, Universiti Malaya	Noraini Talip
*Globba pendula* Roxb.	NTZ11	Rimba Ilmu, Universiti Malaya, Kuala Lumpur	Noraini Talip
	NTZ12	R mba Ilmu, Universiti Malaya, Kuala Lumpur	Noraini Talip
*Globba roxburghiana*	NTZ14	Rimba Ilmu, Universiti Malaya, Kuala Lumpur	Noraini Talip
	NTZ15	Rimba Ilmu, Universiti Malaya, Kuala Lumpur	Noraini Talip
	NTZ16	Nursery of School of Biological Sciences, Universiti Malaya	Noraini Talip
*Globba unifolia* Ridl.	NTZ17	Rimba Ilmu, Universiti Malaya, Kuala Lumpur	Noraini Talip
	NTZ18	Rimba Ilmu, Universiti Malaya, Kuala Lumpur	Noraini Talip
	NTZ19	Rimba Ilmu, Universiti Malaya, Kuala Lumpur	Noraini Talip
*Globba variabilis* Ridl.	NTZ20	Rimba Ilmu, Universiti Malaya, Kuala Lumpur	Noraini Talip
	NTZ21	Rimba Ilmu, Universiti Malaya, Kuala Lumpur	Noraini Talip
	NTZ22	Merapoh Forest Reserve, Pahang	Noraini Talip
*Globba variabilis* var. *pusilla* Lim.	NTZ23	Rimba Ilmu, Universiti Malaya, Kuala Lumpur	Noraini Talip
	NTZ24	Nursery of School of Biological Sciences, Universiti Malaya	Noraini Talip
	NTZ25	Nursery of School of Biological Sciences, Universiti Malaya	Noraini Talip

**Table 2:** Summary of midrib and petiole anatomical characteristics.

Species	Midrib				Petiole					

	Trichomes	Vascular system and the number of vascular bundles			Trichomes	Vascular system and the number of vascular bundles				Outer tissue (no. of cells)
	
		I	II	III		I	II	III	IV	
*G. aurantiaca*	Simple unicellular abaxially	9	8	1	Simple unicellular adaxially and abaxially	11	2	2	8	-
*G. cernua*	Simple unicellular abaxially	6	2	1	Simple unicellular adaxially and abaxially	11	2	1	9	2
*G. leucantha*	Simple unicellular adaxially and abaxially	11	10	2	Simple unicellular adaxially and abaxially	23	6	7	18	-
*G. patens*	Simple unicellular abaxially	7	4	3	Simple unicellular adaxially and abaxially	17	19	3	16	1–2
*G.patens* var. *costulata*	Simple unicellular abaxially	7	2	3	Simple unicellular adaxially and abaxially	23	7	8	19	1–2
*G.pendula*	-	6	2	-	Simple unicellular adaxially and abaxially	12	2	1	6	1
*G.roxburghiana*	-	4	2	1	-	15	4	3	13	1
*G.unifolia*	Simple unicellular abaxially	9	5	1	Simple unicellular adaxially and abaxially	23	30	22	21	2–3
*G.variabilis*	Simple unicellular abaxially	6	2	2	Simple unicellular adaxially and abaxially	16	6	6	9	2
*G.variabilis* var*. pusilla*	Simple unicellular adaxially and abaxially	4	-		Simple unicellular adaxially and abaxially	13	5	3	9	1–2

**Table 3:** Leaf lamina anatomical characteristics.

Species	Epidermis adaxial and abaxial (height:width)	Hypodermis (interrupted/ continuous/ adaxially/ abaxially)	Palisade (no. of layers)	Trichomes	Mesophyll (no. of layers)
*G.aurantiaca*	1:1–1:3; 1:1–1:2	Continuous abaxially	2–3	–	2–3
*G.cernua*	1:1–1:3; 1:1–1:2	Continuous abaxially	2–3	–	2–3
*G.leucantha*	1:1–1:3; 1:1–1:2	Continuous abaxially	2–3	Simple unicellular	4–5
*G.patens*	1:1–1:3; 1:1–1:3	Continuous abaxially	2	Simple unicellular Tufted	2–3
*G.patens* var. *costulata*	1:1–1:2; 1:1–1:2	Continuous abaxially	2–3	Simple unicellular	2–3
*G.pendula*	1:1–1:3; 1:1–1:3	Continuous abaxially	2	Simple unicellular	2–3
*G.roxburghiana*	1:1–1:3; 1:1–1:3	Continuous abaxially	2	–	1–2
*G.unifolia*	1:1–1:3; 1:1–1:3	Continuous abaxially	2	Simple unicellular	1–2
*G.variabilis*	1:1–1:3; 1:1–13	Continuous abaxially	2	Simple unicellular	1–2
*G.variabilis* var*. pusilla*	1:1–1:2; 1:1–1:2	Continuous abaxially	2	Simple unicellular Papillae	1–2

**Table 4:** Dichotomous identification key that includes atomical characteristics for the *Globba* species studied.

No.		
1	Stomata amphistomatic	2
1	Stomata heterostomatic	5
2	Simple trichome on midrib; simple trichome on lamina	3
2	Midrib dan lamina glabrous	*G. roxburghiana*
3	Vascular bundles arranged on midrib arc I and II	*G. pendula*
3	Vascular bundles arranged on midrib arc I, II and III	4
4	Midrib outline on abaxial surface arched or V-shaped	*G. leucantha*
4	Midrib outline on abaxial surface arched or U-shaped	*G. unifolia*
5	Tufted trichome on petiole; simple and tufted trichome on lamina	*G. paten*
5	Simple trichome on petiole; simple trichome or papilliae on lamina	6
6	Vascular bundles arranged on midrib arc I or I and II	7
6	Vascular bundles arranged on midrib arc I, II and II	8
7	Simple trichome and papillae on lamina; tip margin straight	*G. variabilis* var. *pusilla*
7	Simple trichome on lamina; tip margin recurved down	*G. paten* var. *costulata*
8	Fibres at large vascular bundles extent to adaxial epidermis; epidermis abaxial height:width 1:1–1:2	*G.variabilis*
8	Fibres at large vascular bundles not extent to adaxial epidermis; epidermis abaxial height:width 1:1–1:3	9
9	Vascular system and the number of petiole vascular bundles arcs I, II, III and IV ratio 11:2:2:8	*G. aurantiaca*
9	Vascular system and the number of petiole vascular bundles arcs I, II, III and IV ratio 23:6:7:13	*G. cernua*
